# A Model for Improving the Learning Curves of Artificial Neural Networks

**DOI:** 10.1371/journal.pone.0149874

**Published:** 2016-02-22

**Authors:** Roberto L. S. Monteiro, Tereza Kelly G. Carneiro, José Roberto A. Fontoura, Valéria L. da Silva, Marcelo A. Moret, Hernane Borges de Barros Pereira

**Affiliations:** 1 Programa de Modelagem Computational, SENAI CIMATEC, Av. Orlando Gomes 1845, Salvador, 41.650-010, Brazil; 2 Universidade do Estado da Bahia, Salvador, Brasil; 3 Universidade Estadual de Ciências da Saúde de Alagoas, Maceió, Brazil; Beihang University, CHINA

## Abstract

In this article, the performance of a hybrid artificial neural network (i.e. scale-free and small-world) was analyzed and its learning curve compared to three other topologies: random, scale-free and small-world, as well as to the chemotaxis neural network of the nematode Caenorhabditis Elegans. One hundred equivalent networks (same number of vertices and average degree) for each topology were generated and each was trained for one thousand epochs. After comparing the mean learning curves of each network topology with the C. elegans neural network, we found that the networks that exhibited preferential attachment exhibited the best learning curves.

## Introduction

Emmert-Streib [1] demonstrated the effect of topology on the performance of neural networks. they compared the performances of random-topology networks [2], scale-free networks [3], and small-world networks [4]. Bohland and Minai [5] highlighted that small-world networks are more economical because these networks have fewer connections and perform as fast as denser networks when applied to associative memory systems.

Watts and Strogatz [4] analyzed the properties (mean shortest path and mean clustering coefficient) of the neural network of the nematode *Caenorhabditis elegans*[6, 7] and found that this network exhibits small-work network characteristics. Latora and Marchiori [8] also reached this conclusion when analyzing the efficiency of the neural network of *C. elegans*. Chen et al. [9] also studied the efficiency of this network and argued that this characteristic is an evolutionary trait.

Although the aforementioned authors classified the neural network of *C. elegans* as a small-world network, Morita et al. [10] argued that the Watts and Strogatz [4] model is insufficient to explain its properties. It should be emphasized that *C. elegans* was used as a benchmark for these studies because it is the only animal whose neural network has been fully mapped and is used as a model for various studies involving neurodegeneration and neuroplasticity (e.g., [11–13]).

**Fig 1:**
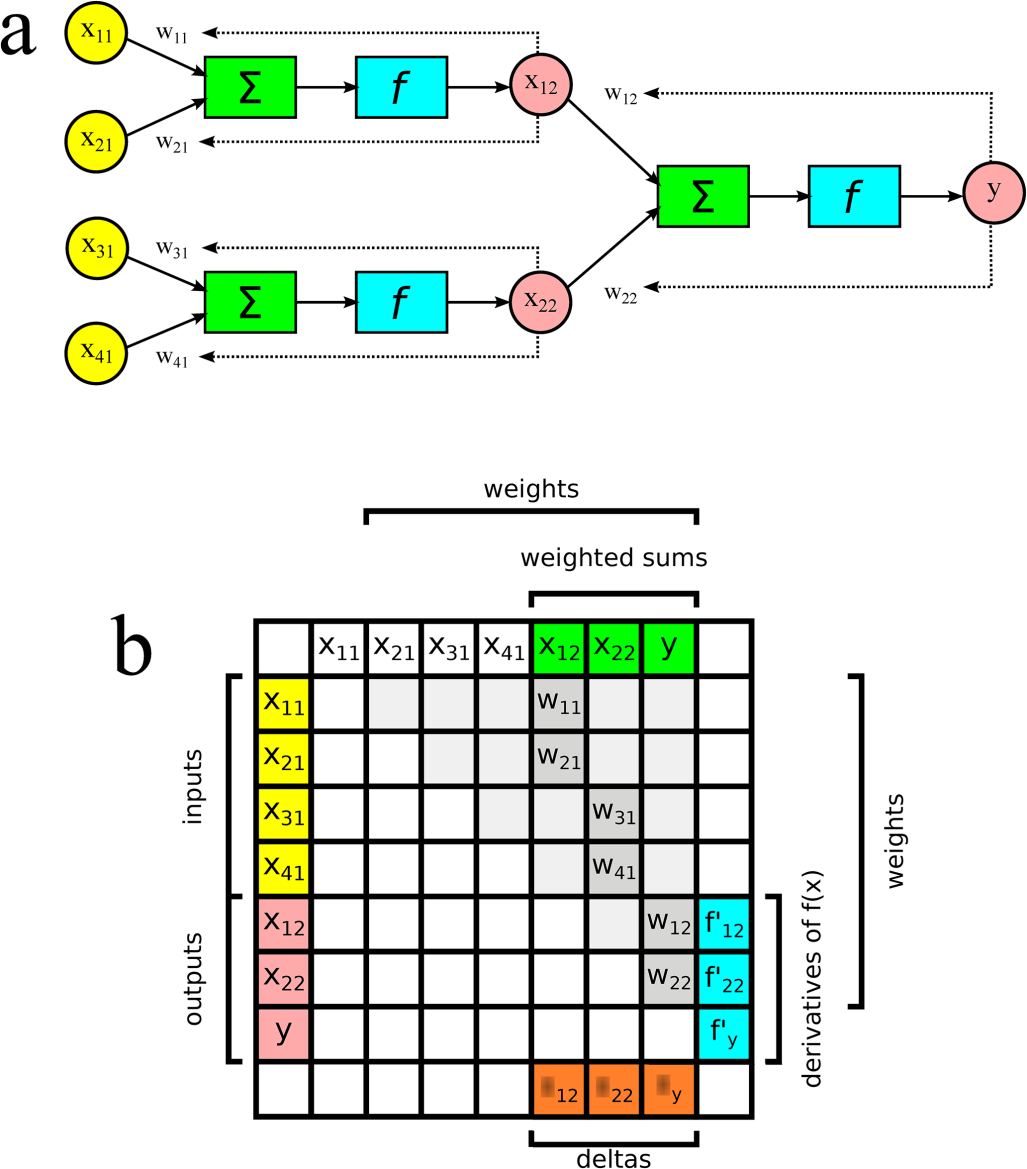
Artificial neural network and its learning matrix. a) Two-layer perceptron based on Rosenblatt [21] and Nazzal et al. [22]. b) Learning matrix elements.

These studies utilized simplified models to simulate the neural network of the animal. In this paper, we proposed a method that allows the original neural network of the animal (represented by an augmented adjacency matrix, called learning matrix in this article, Fig 1b) to be trained and compares its performance with random, small-world, scale-free and hybrid topology networks.

Furthermore, we compare the learning curves of four network topologies (random [2], scale-free [3], small-world [4], and hybrid [14, 15]) with the performance of the neural network for chemotaxis in *C. elegans*[16–19]. This is the first time, to the best of our knowledge, that a comparative analysis of the performance of a hybrid neural network was done.

## Materials and Methods

We selected a sub-network of the main component of the neural network of *C. elegans* to perform this study: the chemotaxis network. This network, studied by Ward [16], Segev and Ben-Jacob [18], Pierce-Shimomura et al. [17], and Dunn et al. [19], among others, consists of 15 neurons that are interconnected by chemical and electrical synapses (there are two pairs of each neuron; thus, two identical networks are formed for chemotaxis). In this study, we made no distinction between chemical and electrical synapses and only used one neuron from each pair to simplify modeling. This simplification does not lead to any loss of information, since we investigate the efficiency of the topological structure of the neural network regarding the flow of information in terms of learning correctness and epochs. Simulation results of the C. elegans network with the electrical synapses removed validate this assumption and are shown in S1 Appendix.

**Fig 2:**
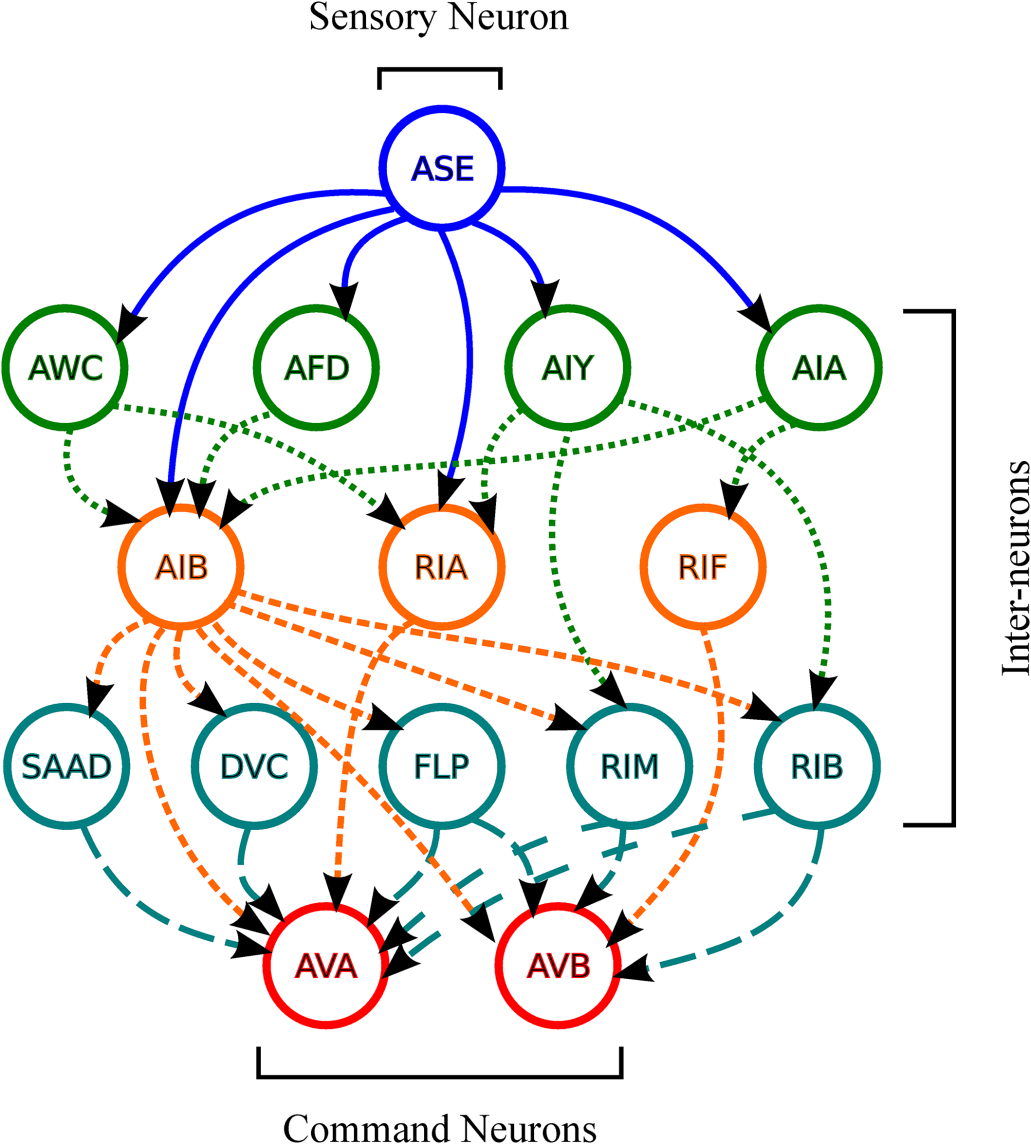
The chemotaxis neural network of *C. elegans*, based on [6, 7, 19]. The line type identifies the arcs leaving the neurons (vertices) of each layer. The solid line arcs leave the ASE sensory neuron; the dotted line arcs leave the AWC, AFD, AIY and AIA interneurons; the short dashed line arcs leave the AIB, RIA and RIF interneurons; and the dashed line arcs leave the SAAD, DVC, FLP, RIM and RIB interneurons.

The model introduced by Dunn et al. [19] contains one input neuron, ASE, and one output neuron, which combines neurons AVA and AVB into a single neuron. We chose to treat the two neurons separately in this study. So, we drawn the directed graph shown in Fig 2. This network is similar to that presented by [19], except that the loops have been removed and the chemical and electrical synapses are represented by a single oriented line segment. [19] and Varshney et al. [7]

Based on this graph, we created 100 equivalent artificial networks (same number of vertices and average degree) of each topology: 100 random networks, 100 scale-free networks, 100 small-world networks, and 100 hybrid networks. The random, scale-free, and small-world networks were created using algorithms adapted from Batagelj and Brandes [20]. To create the hybrid networks, we initially created small-world networks with the same number of vertices and an initial average degree slightly smaller than the one for the *C. elegans* (the initial average degree is obtained empirically by starting with a value of one or two units lower than desired and increasing this value in 0.1 steps to obtain a network with the average degree nearest the desired); then, new edges were added to the networks according to the probability $p_{i}=\frac{k_{i}}{n_{edges}}$, where *k*_*i*_ is the vertex degree, and *n*_*edges*_ is the number of edges existing in the network. Barabási and Albert [3] proposed $p_{i}=\frac{k_{i}}{\sum_{j=1}^{n}k_{j}}$ for preferential attachment. However, this formula results in an extremely small number of preferential connections given the small size of the network. These networks were saved in Pajek format files for subsequent use in the simulations.

Each network was trained 1000 times or until learning reached 100% using a set of 100 pairs of input and output values, which correspond to the rules shown in Table 1. This table was defined based on the analysis of the experiment conducted by Dunn et al. [19]. The ASE value corresponds to the variation in the *NH*_4_ *Cl* concentration detected by this neuron, which is expressed as 10^−3^ *mM*/*s*. We set the value of ±5 × 10^−3^ *mM*/*s* for the lower and upper limits of the variation range of *NH*_4_ *Cl* concentration based on the analysis of the graphs shown in Dunn et al. [19]. This value is an approximation required for our simulations.

**Table 1:** Rules for the simulation of chemotaxis in *C. elegans* based on Dunn et al. [19].

ASE	AVA	AVB
*dC*/*dt* < −5	0	1
−5 ≤ *dC*/*dt* ≤ 5	0	0
*dC*/*dt* > 5	1	0

The mathematical model that was used to construct the artificial neural networks was based on the perceptron created by Rosenblatt [21] and generalized for multiple layers by Nazzal et al. [22]. Fig 1a shows a two-layer perceptron. The perceptron is an easily implemented artificial neuron. However, code development for the construction of an artificial neural network becomes laborious as the number of layers increases. To facilitate our study, we developed an algorithm that enables training and running a neural network using a learning matrix, which is constructed based on the adjacency matrix of the network. Fig 1b shows the learning matrix elements for the two-layer perceptron in Fig 1a. These algorithms are presented in detail in S2 Appendix.

A perceptron consists of four elements: input signals, adder, activation function, and output signal. Multi-layer perceptrons consist of several artificial neurons arranged in layers, wherein a neuron output is the neuron input of the next layer. Feedback, wherein a neuron output returns to the same layer, may exist if necessary. Our experiment used five-layer perceptrons without feedback, wherein the first layer (input) consisted of the ASE neuron; the second layer consisted of the AWC, AFD, AIY, and AIA neurons; the third layer consisted of the AIB, RIA, and RIF neurons; the fourth layer consisted of the SAAD, DVC, FLP, RIM, and RIB neurons, and the fifth layer (output) consisted of the AVA and AVB neurons.

To calculate the output value of a neuron, we used the function *y*_*j*_ = *f*(*x*_*j*_), where *f*(*x*_*j*_) is the neuron activation function, and *x*_*j*_ is the value of the weighted sum of the inputs in this neuron defined by $x_{j}=\sum_{i=1,j=1}^{n}x_{i}\cdot w_{i,j}$, where *x*_*i*_ is the input value at synapse *i* of neuron *j*, and *w*_*i*,*j*_ is the weight of this synapse. We chose the sigmoid function, $f\left(x_{j}\right)=\frac{1}{1+e^{-x_{j}}}$, as the activation function because this function is commonly used to simulate the output signal of neurons in *C. elegans* (e.g., [19]).

The process of training a perceptron, be it a single layer or multiple layers, occurs by adjusting the weights of the neural synapses. For this purpose, we used Eq 1, which is based on the study by Nazzal et al. [22].

$$\begin{array}{c} w_{i,j}=w_{i,j}\cdot \eta \cdot \delta_{j}\cdot f^{\prime }\left(x_{j}\right)\cdot x_{i} \end{array}$$ (1)

where *η* is a real number between 0 and 1. We used *η* = 0.45 in our study after testing various values between 0.05 and 0.95 with 0.05 increments.

To calculate *δ*_*j*_, the output error of neuron *j*, we used two equations, *δ*_*j*_ = *z*_*j*_ − *y*_*j*_, for the weights of the last layer, and $\delta_{j}=\sum_{i=1,j=n}^{i=n,j=1}w_{i,j}\cdot \delta_{i}$, for the weights of the intermediate layers. In this formulas *z*_*j*_ is the expected output value of neuron *j* and *δ*_*i*_ is the error value of input neuron *i* of the layer after neuron *j*. Furthermore, $f^{\prime }\left(x_{j}\right)=\frac{1}{1+e^{-x_{j}}}\cdot \left(1-\frac{1}{1+e^{-x_{j}}}\right)$ is the derivative of the activation function of neuron *j*.

For each network, we ran the algorithm 1000 times and saved the hit percentage of the input and output set at each epoch.

The algorithms described in S2 Appendix were developed in order to facilitate performing simulations using neural networks with complex topologies, such as those studied herein. These algorithms were implemented in the programming language GuaráScript, which we also designed to facilitate the construction of scientific applications. All software programs that were used as the basis for this study are available for download on the GuaráScript project website: http://www.guarascript.org.

The entire dataset used to perform the simulations are included in S1 Dataset.

## Results

The results were divided into two groups. In group 1, we have considered only those simulations where there was 100% of learning. In group 2, we have considered all the simulations, even when there was less than 100% of learning.

**Fig 3:**
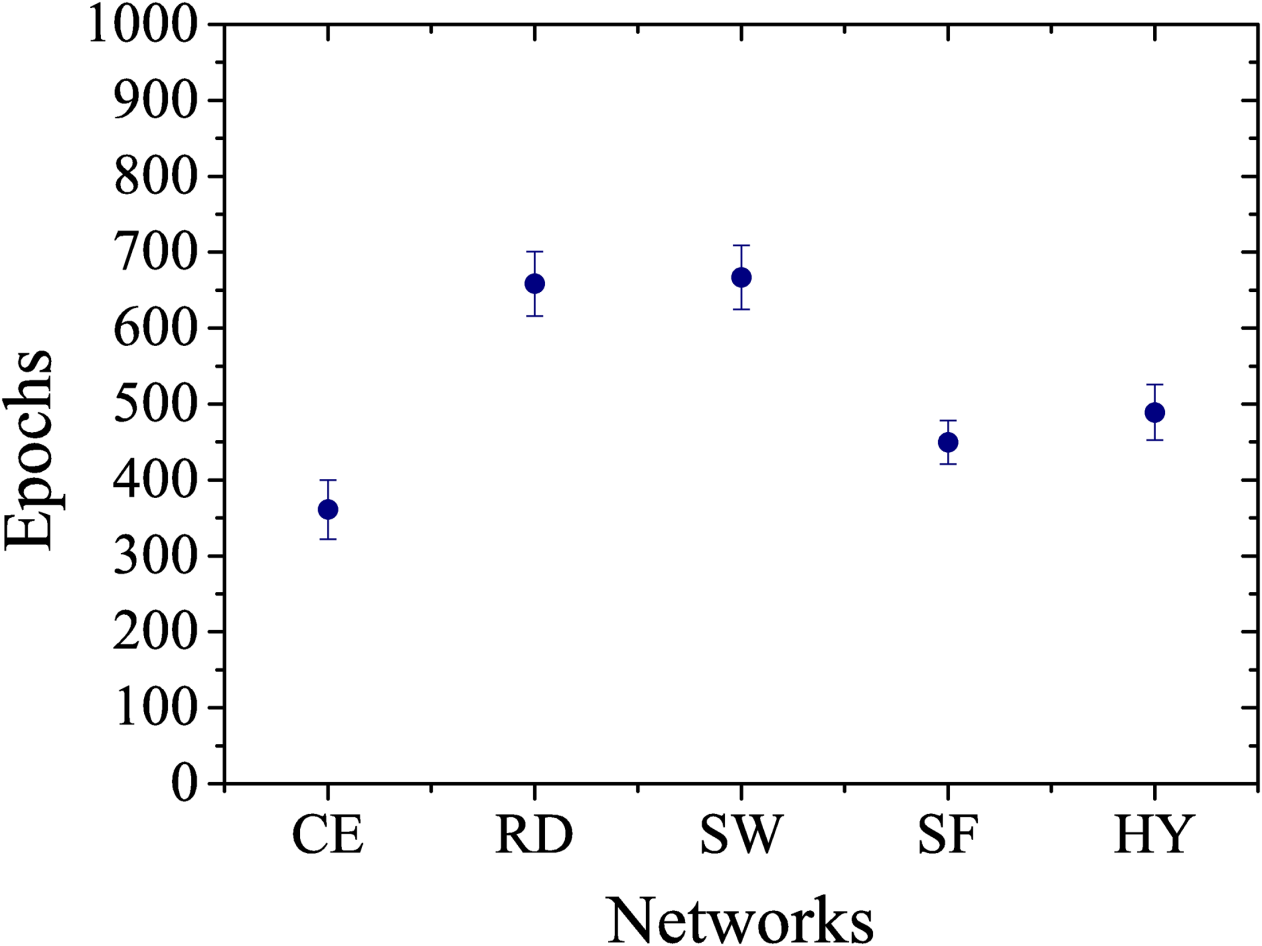
The number of epochs. (CE) *C. elegans*, (RD) random, (SW) small-world, (SF) scale-free and (HY) hybrid networks necessary to learn to interpret 100 input signals. Mean of 100 samples.

**Fig 4:**
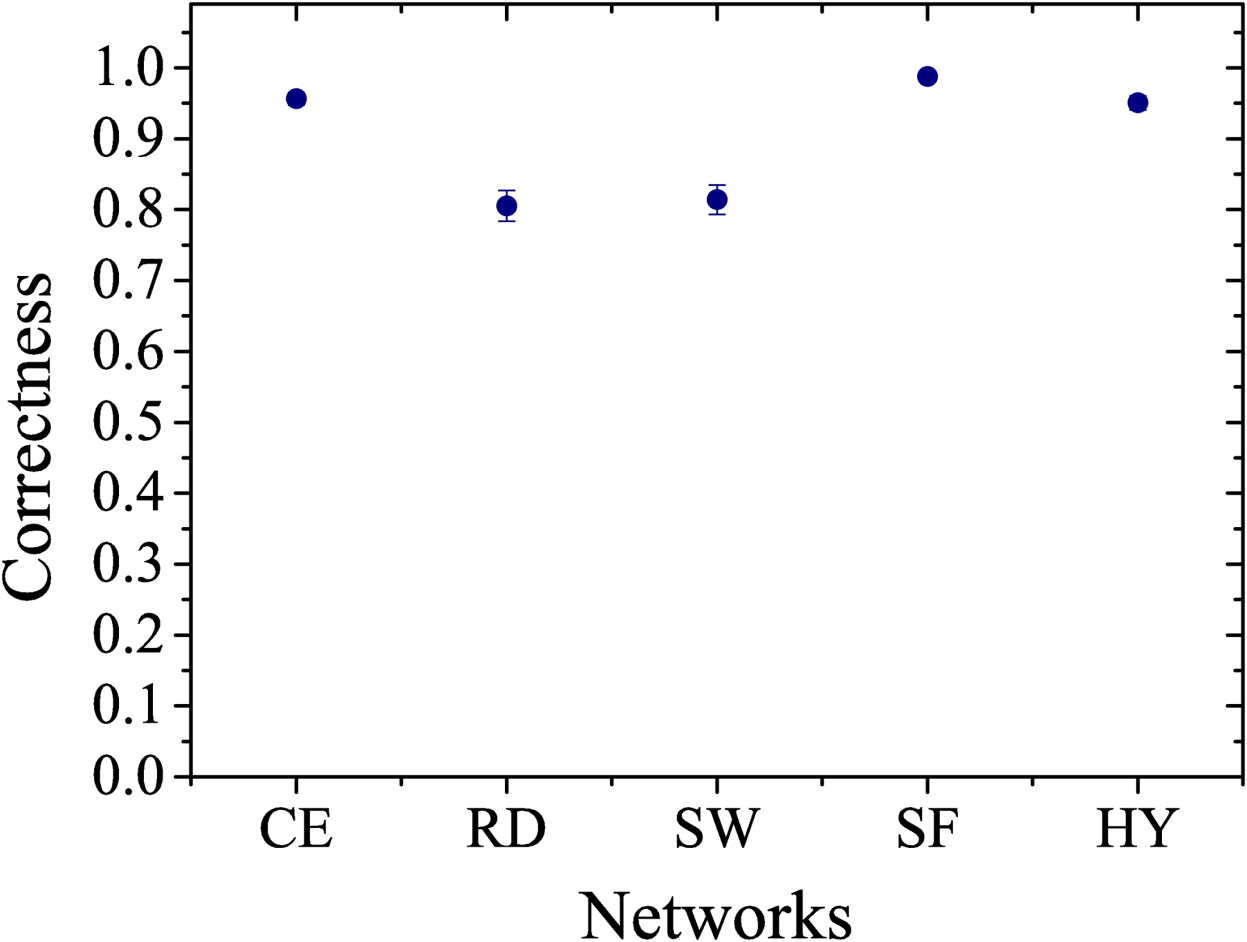
The correctness. (CE) *C. elegans*, (RD) random, (SW) small-world, (SF) scale-free and (HY) hybrid networks, when attempting to learn to interpret 100 input signals. Mean of 100 samples.

Considering the simulations of group 2, Figs 3 and 4 show the results of the simulations performed with 400 artificial neural networks, composed by 100 random, 100 scale-free, 100 small-world and 100 hybrid (scale-free and small-world) networks, in terms of the number of epochs and correctness of the networks, comparing them with the values obtained from the original network of *C. elegans*. Fig 3 shows that the networks with preferential attachment (i.e. free-scale and hybrid) learn more rapidly than the networks where those characteristics are not present (i.e. random and small-world). In Fig 4, we observe that the network with hybrid topology has a mean number of epochs to learn that is close to the *C. elegans* network. These results provide evidence that the neural network of *C. elegans* can have an hybrid topology with characteristics of scale-free and small-world networks, reinforcing the observations made by [10].

**Fig 5:**
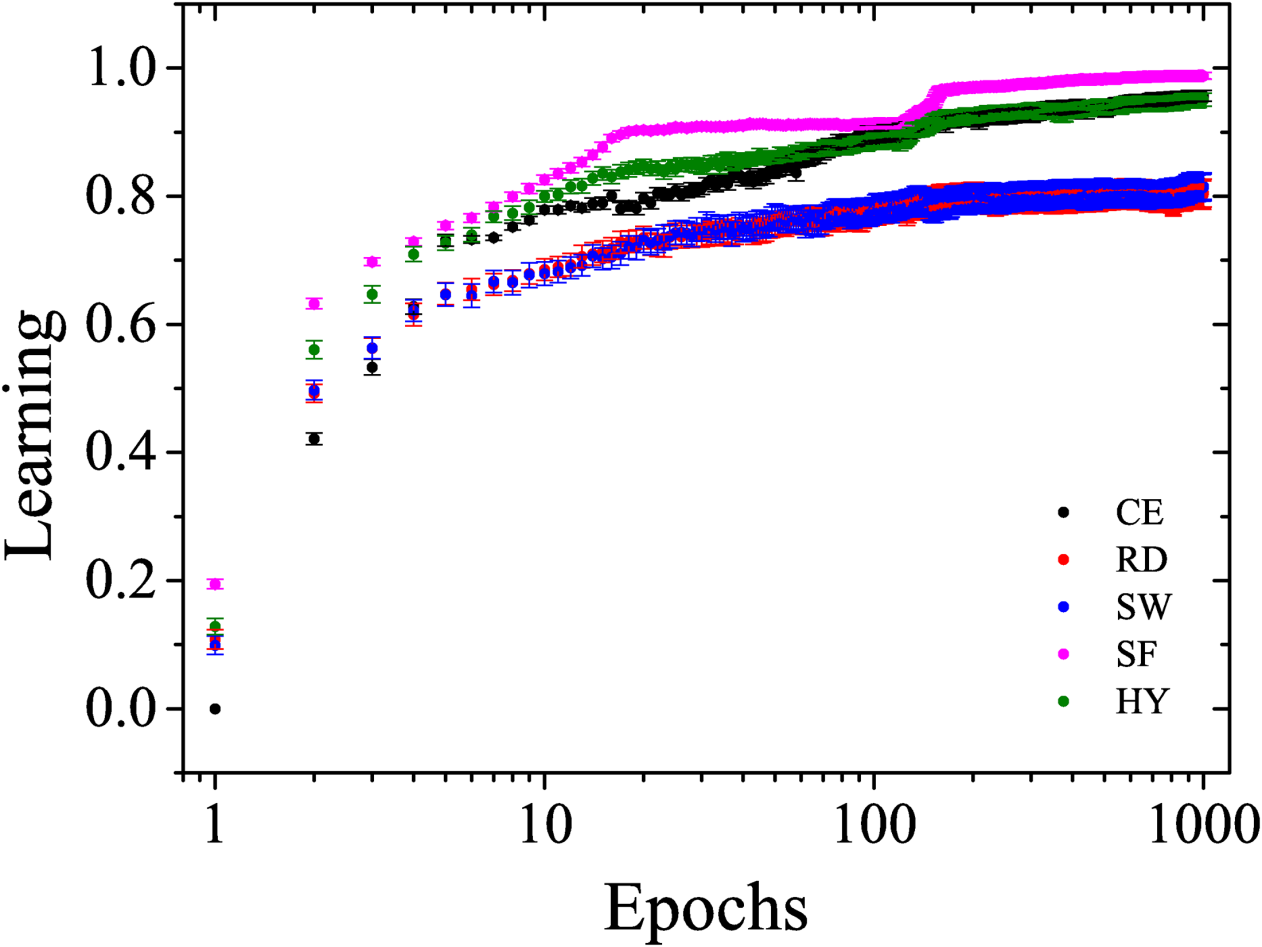
Learning curves of the neural network of *C. elegans* and the random, small-world, scale-free and hybrid artificial neural networks.

Fig 5 compares the mean learning curves of the *C. elegans*, hybrid, random, scale-free, and small-world networks. The scale-free, hybrid, and *C. elegans* networks learned faster than the random and small-world networks. Conversely, the learning curve of the animal neural network approaches the hybrid neural network at approximately the hundredth epoch.

Considering that although the neural network of *C. elegans* has characteristics of a small-world network [23], its properties may not be explained using only this model [10]. Furthermore, Chatterjee and Sinha [24] argued that there is a correlation between the degree centrality of the neurons of the *C. elegans* network and its neurological importance, which characterizes the preferential attachment. Within this context, we have evidence that this network has the characteristics of a scale-free network, and the networks where preferential attachment occurred were those that exhibited the best learning curves.

As observed in Fig 5, there is an evidence that the neural network of the animal exhibits an hybrid neural network (i.e. small-world and scale-free properties).

We also noticed that the theoretical hybrid network behaved like the original network of the animal, in terms of its ability to properly learn (correctness) the rules imposed on the model (Fig 4).

In order to validate our conclusion, we performed a similar experiment using a semantic network for controlling a gas sniffer robot. The results are similar to the ones obtained with the C. Elegans (i.e. networks with preferential attachment have better learning curves). More details on this experiment are presented in S3 Appendix.

## Conclusions

In this study, we analyzed the performance of four network topologies, including random, small-world, scale-free and hybrid. These topologies were used to compare their results with the results of the neural network of *C. elegans*. Further, we presented two algorithms that were suitable for the implementation of artificial neural networks with complex topologies (random, small-world, scale-free and hybrid).

We compared the learning curves of four different network topologies that are used in modeling artificial neural networks. We observed that the scale-free, hybrid, and *C. elegans* networks learned faster than the other topologies because they displayed preferential attachment.

We used the neural network for chemotaxis in the nematode *Caenorhabditis elegans* as the benchmark and found that near the hundredth epoch, its learning curve distances itself from the random and small-world networks and approaches the hybrid network curve. This result provides evidence that the neural network of the animal exhibits an hybrid neural network (i.e. small-world and scale-free properties).

When analyzing the structure and function of the neural network of *C. elegans*, [9] emphasized that the network is highly optimized and that this optimization is an evolutionary trait. This hypothesis is reinforced by the results observed in Figs 3 and 4, which show that the random and small-world networks have the lowest correctness and the worst time to learn, while the scale-free network features 100% correctness.

On the other hand, when studying the efficiency of the neural network of *C. elegans*, [8] emphasized that the neural network behaves as a small-world network and that this type of network has the property of being highly resistant to failure. Thus, it is natural that in its evolutionary process, the animal has experienced various network topologies and that natural selection has favored individuals with extremely fast learning, accuracy in their responses and the ability to withstand failures in its neurological structure (e.g., diseases and injuries caused by predators).

In fact, in addition to displaying characteristics of a small-world network, the neural network of *C. elegans* has other properties that suggest that this network may also behave as a scale-free network, i.e., a hybrid network.

## Supporting Information

S1 Appendix Results of the simulation after removal of the electrical synapses (gap junctions) of the C. elegans chemotaxis neural network. (PDF)

S2 Appendix Training Process Summary and Algorithms. (PDF)

S3 Appendix Simulation results of a gas sniffer robot. (PDF)

S1 Dataset Complete dataset. (ZIP)
