# Supplementary material for: A Model for Improving the Learning Curves of Artificial Neural Networks
Source: PLoS One. 2016 Feb 22;11(2):e0149874. doi: 10.1371/journal.pone.0149874 (PMC4763452; doi:10.1371/journal.pone.0149874)
Supplement: S1 Appendix — (PDF) [file pone.0149874.s001.pdf]

Results of the simulation after removal of the electrical synapses (gap junctions) of the C. elegans chemotaxis neural network

Roberto L. S. Monteiro,
Tereza Kelly G. Carneiro,
José Roberto A. Fontoura,
Valéria L. da Silva,
Marcelo A. Moret,
Hernane Borges de Barros Pereira

February 11, 2016

We selected a sub-network of the main component of the neural network of *C. elegans* to perform this study: the chemotaxis network. This network, studied by Ward [1], Segev and Ben-Jacob [3], Pierce-Shimomura et al. [2], and Dunn et al. [4], among others, consists of 15 neurons that are interconnected by chemical and electrical synapses (there are two pairs of each neuron; thus, two identical networks are formed for chemotaxis). Chemical synapses are unidirectional and the signal direction can be detected through electromyography (Varshney, 2011) while electrical synapses are bidirectional, faster and have lower gain. Electrical synapses are found in systems that require a fast response, having also a role in the synchronization of a group of neurons. In our model the electrical synapses are very few.

In the article, we made no distinction between chemical and electrical synapses and only used one neuron from each pair to simplify modeling. This simplification does not lead to any loss of information, since we investigate the efficiency of the topological structure of the neural network regarding the flow of information in terms of learning correctness and epochs. To validate that, we modeled the C. elegans network without the electrical synapses and obtained similar results. The removed connections are: AFD-AIB, AIY-RIM, AIB-DVC, AIB-FLP e RIB-AVB.

Figure 1 presents the learning curves of the neural network of C. Elegans network with and without the gap junctions (eletrical synapes).

Figure 1. Learning curves of the neural network of C. Elegans network with (CE) and without the gap junctions (eletrical synapes) (CEOnlyCh).

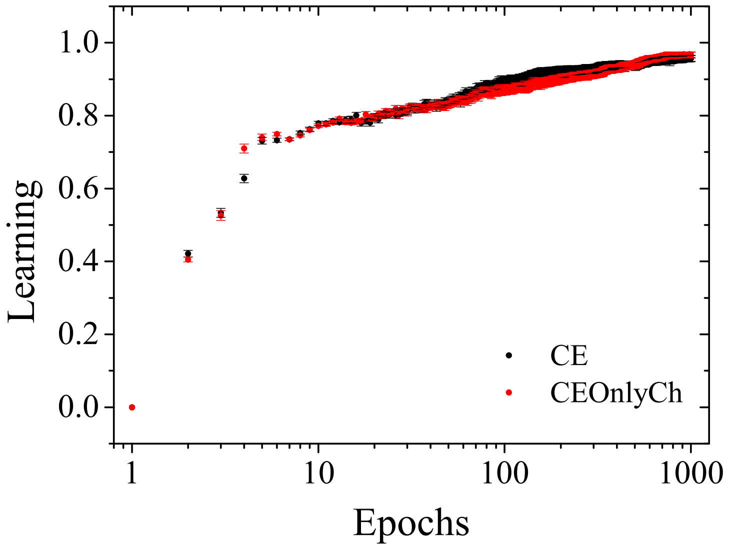

References

[1] Ward S. Chemotaxis by the Nematode Caenorhabditis elegans: Identification of Attractants and Analysis of the Response by Use of Mutants. Proceedings of the National Academy of Sciences. 1973;70(3):817–821.

[2] Pierce-Shimomura JT, Morse TM, Lockery SR. The fundamental role of pirouettes in Caenorhabditis elegans chemotaxis. Journal of Neuroscience. 1999;19(21):9557–9569.

[3] Segev R, Ben-Jacob E. Generic modeling of chemotactic based self-wiring of neural networks. Neural Networks. 2000;13(2):185–199.

[4] Dunn N, Lockery S, Pierce-Shimomura J, Conery J. A Neural Network Model of Chemotaxis Predicts Functions of Synaptic Connections in the Nematode Caenorhabditis elegans. Journal of Computational Neuroscience. 2004;17(2):137–147.
