# Supplementary material for: A Model for Improving the Learning Curves of Artificial Neural Networks
Source: PLoS One. 2016 Feb 22;11(2):e0149874. doi: 10.1371/journal.pone.0149874 (PMC4763452; doi:10.1371/journal.pone.0149874)
Supplement: S2 Appendix — (PDF) [file pone.0149874.s002.pdf]

Training Process Summary and Algorithms

Roberto L. S. Monteiro,
Tereza Kelly G. Carneiro,
José Roberto A. Fontoura,
Valéria L. da Silva,
Marcelo A. Moret,
Hernane Borges de Barros Pereira

February 11, 2016

Figs. 1a, 1b, 1c, 1d, 1e and 1f (presented here) illustrate the training process of the neural network in Fig. 2a (presented in article) using a learning matrix (Fig. 2b presented in article); the steps for both algorithms 1 and 2 are summarized below:

1. *Learning matrix initialization*: first, load the learning matrix using the values of the adjacency matrix of the network. The names of the neurons in the first row and first column are replaced by zeros (0);

2. *Assignment of random weights to the neural synapses*: random values between 0 and 1 are assigned to each cell with a value of 1 between the second and one before last row and the second and one before last column;

3. *Assignment of input values*: the values of the input signals are copied to the learning matrix in the first row in the positions corresponding to the neurons of the first layer. Fig.1b shows the state of the learning matrix at this point of the algorithm (matrix cells affected in this step are highlighted on the left side of the figure) This process is represented by lines 1 to 4 of Algorithm 1;

4. *Calculation of the weighted sums of input signals*: the sum of the input values, which is multiplied by the weights of the corresponding neural synapses, is calculated for each neuron. These sums are stored in the first row of the matrix. Fig.1c shows the state of the learning matrix after calculating the weighted sums (the matrix cells affected in this step are highlighted on the upper right side of the figure). The movement in this step when looping through the matrix occurs from the left to the right and from top to bottom. This process is represented by lines 5 to 15 of Algorithm 1;

5. *Calculation of the output values of the neural network*: the output values of the neural network are obtained by calculating the activation function for the values of the weighted sums obtained in the previous step and stored in the first row. The results are stored in the first column in the row corresponding to each neuron. The values of the derivatives of the

activation function are also calculated, and these results are stored in the last column in the row corresponding to each neuron. Fig.1d shows the state of the learning matrix after calculating the neuron outputs (the cells affected in the matrix are highlighted on the left and right sides of the figure). This process is represented by lines 16 to 19 of Algorithm 1;

6. *Calculation of the deltas for adjusting the weights*: the deltas for adjusting the weights in the last layer are calculated by subtracting the output value obtained from the expected values. For the neurons in the intermediate layers, the delta value of an output neuron j is calculated by adding the values of the deltas of input neuron i following j, which is weighted by the weights of synapses between i and j. This result is stored in the last row of the matrix in the position corresponding to neuron j. Fig.1e shows the state of the learning matrix after calculating the deltas of the neuron outputs (the cells affected in the matrix are highlighted on the lower right side of the figure). The movement in this step when looping through the matrix occurs from the right to the left and from top to bottom. This process is represented by lines 21 to 36 of Algorithm 1;

7. *Adjustment of the weights of the neural synapses*: the weights of the neural synapses are adjusted using Equation 1 (presented in article) based on the deltas and the derivatives of the activation functions calculated previously. Fig.1f shows the state of the learning matrix after calculating the adjustment of the weights of the neural synapses (the cells affected in the matrix are highlighted in the center of the figure). The movement in this step when looping through the matrix occurs from the left to the right and from top to bottom. This process is represented by lines 37 to 47 of Algorithm 1;

8. *Sets the network to the training set*: perform steps 3 to 7 until all the inputs and outputs present in the training set have been processed;

9. *Train the network*: proceed to step 3 until the maximum number of epochs is reached or until learning reaches 100%. In this study, we saved the hit percentage obtained in each epoch to later plot the graph of the learning curve.

This algorithm may be easily implemented using nested loops and requires a storage space of $(n+2)^2$ multiplied by the size of the real number of the machine used. A trained network may be used to analyze the set of input signals using the method shown by running steps 1 to 5 and excluding the calculation of the derivatives of the activation functions, which is only necessary in the learning process. Algorithm 2 is used to process the signals that are provided to the neural network.

Figure 1. Interactive learning process. a) learning matrix initialization; b) assignment of the input signals of the neural network to the input cells of the learning matrix; c) calculation of the weighted sums of input signals of the neural network layers; d) calculation of the output signals of the neural network and of the derivatives of the activation functions; e) calculation of the deltas to adjust the weights of the neural synapses; f) adjustment of the weights of the neural synapses.

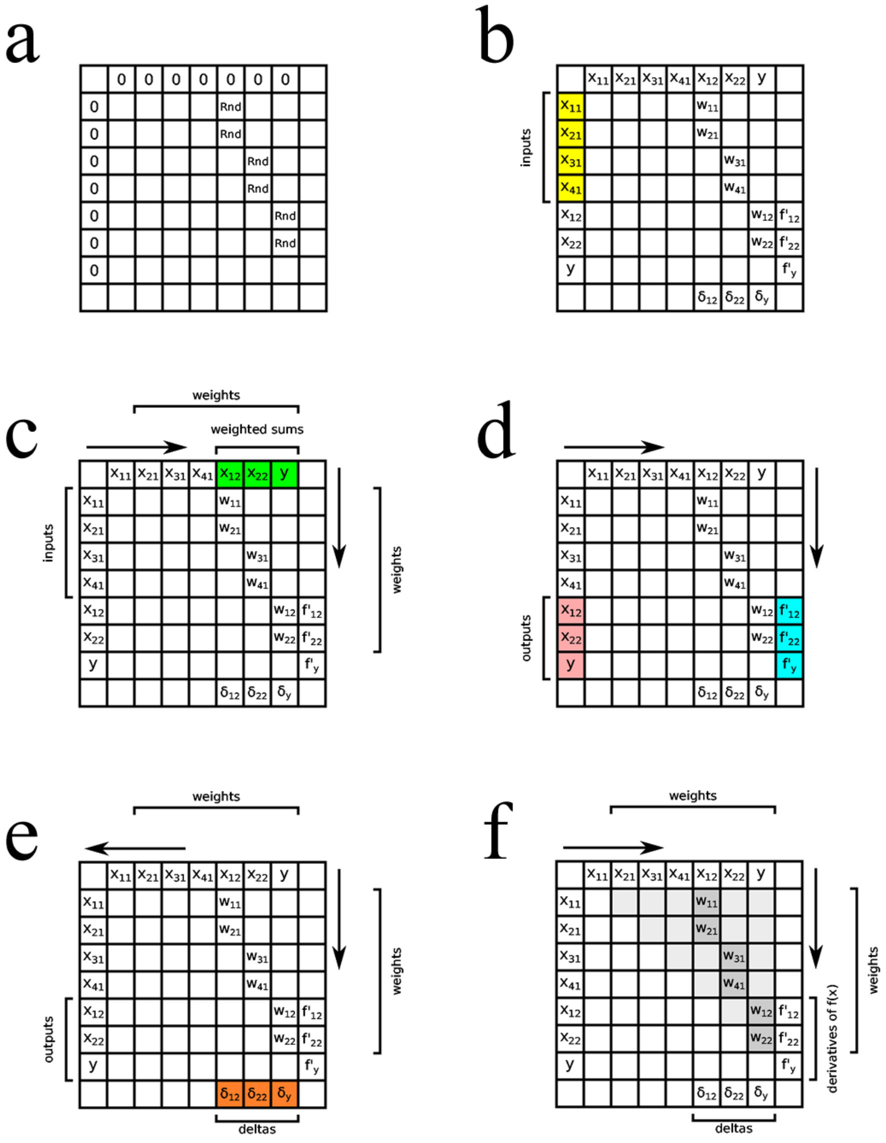

Algorithm 1 Training Process

Require: $net_{i,j} \leftarrow$ matrix with the weights of each neural synapse, $in_j \leftarrow$ input data for learning, $out_j \leftarrow$ output data for learning, $n_{inputs} \leftarrow$ number of input neurons in the first layer, $n_{outputs} \leftarrow$ number of output neurons in the last layer, $dimension_i \leftarrow$ number of rows in the matrix of neural synapses, $dimension_j \leftarrow$ number of columns in the matrix of neural synapses

{Assigns input signals}
for $j \leftarrow 1$ to $(n_{inputs} - 1)$ **do**
  $net_{0,j} \leftarrow in_j$
end for
{Calculates the neurons outputs}
for $j \leftarrow (n_{inputs} + 1)$ to $(dimension_j - 2)$ **do**
  $net_{0,j} \leftarrow 0$
  for $i \leftarrow 1$ to $(dimension_i - 2 - n_{outputs})$ **do**
    if $i = j$ **then**
      break
    end if
    if $net_{i,j} \neq 0$ **then**
      $net_{0,j} \leftarrow net_{0,j} + net_{i,j} \cdot net_{i,0}$
    end if
  end for
  {Calculates $f(x)$}
  $net_{j,0} \leftarrow \frac{1}{1+e^{-net_{0,j}}}$
  {Calculates $f'(x)$}
  $net_{j,dimension_j-1} = net_{j,0} \cdot (1 - net_{j,0})$
end for
{Calculates delta for the output neurons}
$first = dimension_j - 1 - n_{outputs}$
for $i \leftarrow 0$ to $(n_{outputs} - 1)$ **do**
  $net_{dimension_i-1,first+i} = out_i - net_{first+i,0}$
end for
{Calculates delta for hidden neurons}
for $j \leftarrow (dimension_j - 2)$ down to $(n_{inputs} + 1)$ **do**
  for $i \leftarrow 1$ to $(dimension_i - 2 - n_{outputs})$ **do**
    if $i = j$ **then**
      break
    end if
    if $net_{i,j} \neq 0$ **then**
      $net_{dimension_i-1,i} \leftarrow net_{dimension_i-1,i} + net_{i,j} \cdot net_{dimension_i-1,j}$
    end if
  end for
end for
{Adjusts weights}
for $j \leftarrow (n_{outputs} + 1)$ down to $(dimension_j - 2)$ **do**
  for $i \leftarrow 1$ to $(dimension_i - 2 - n_{outputs})$ **do**
    if $i = j$ **then**
      break
    end if
    if $net_{i,j} \neq 0$ **then**
      $net_{i,j} \leftarrow net_{i,j} + 0.45 \cdot net_{dimension_i-1,j} \cdot net_{j,dimension_j-1} \cdot net_{i,0}$
    end if
  end for
end for
return $net_{i,j}$

Algorithm 2 Signal Processing

Require: $net_{i,j} \leftarrow$ matrix with the weights of each neural synapse, $in_j \leftarrow$ input data for learning, $n_{inputs} \leftarrow$ number of inputs of the neurons of the first layer, $n_{outputs} \leftarrow$ number of output neurons in the last layer, $dimension_i \leftarrow$ number of rows in the matrix of neural synapses, $dimension_j \leftarrow$ number of columns in the matrix of neural synapses

{Creates the output matrix}
$out_{n_{outputs}} \leftarrow zeromatrix$
{Assigns input signals}
for $j \leftarrow 1$ to $(n_{inputs} - 1)$ **do**
    $net_{0,j} \leftarrow in_j$
end for
{Calculates the neurons outputs}
for $j \leftarrow (n_{inputs} + 1)$ to $(dimension_j - 2)$ **do**
    $net_{0,j} \leftarrow 0$
    for $i \leftarrow 1$ to $(dimension_i - 2 - n_{outputs})$ **do**
        if $i = j$ **then**
            break
        end if
        if $net_{i,j} \neq 0$ **then**
            $net_{0,j} \leftarrow net_{0,j} + net_{i,j} \cdot net_{i,0}$
        end if
    end for
    {Calculates $f(x)$}
    $net_{j,0} \leftarrow \frac{1}{1+e^{-net_{0,j}}}$
end for
return $out_{n_{outputs}}$
