# Supplementary material for: A Model for Improving the Learning Curves of Artificial Neural Networks
Source: PLoS One. 2016 Feb 22;11(2):e0149874. doi: 10.1371/journal.pone.0149874 (PMC4763452; doi:10.1371/journal.pone.0149874)
Supplement: S3 Appendix — (PDF) [file pone.0149874.s003.pdf]

Simulation results of a gas sniffer robot

Roberto L. S. Monteiro,
Tereza Kelly G. Carneiro,
José Roberto A. Fontoura,
Valéria L. da Silva,
Marcelo A. Moret,
Hernane Borges de Barros Pereira

February 11, 2016

In order to validate the conclusions presented in the article, we performed an experiment using a semantic network for controlling a gas sniffer robot. The results were similar to the ones obtained with the C. Elegans: networks with preferential attachment have better learning curves.

The simulated robot has a total of 28 neurons, with 2 at the input and 2 at the output. The input neurons receive the gas sensor signals, whose values can vary from 0 to 63, with 0 corresponding to absence of gas and 63 the maximum gas concentration. The output neurons activate two actuators (motors), one at the left and the other at the right of the robot. Table 1 presents the rule taught to the robot neural network.

The robot neural network was created using a semantic network based on the three Asimov robotic rules [1] and trained for one thousand epochs, using a training set with 200 input/output pairs (input values between 0 and 63 and output values between 1 and 0. Figures 1 and 2 shows the simulation results.

Table 1. Simulation rules for a gas sniffer robot. (LS) left sensor, (RS) right sensor, (LA) left actuator, (RA) right actuator.

LS and RS	LA	RA
$LS < RS$	1	0
$LS > RS$	0	1
$LS = RS$	1	1
$LS = 0\ e\ RS = 0$	0	0

Figure 1. The correctness. (SNRR) semantic network based on the Rules of Robotic, (RD) random, (SW) small-world, (SF) scale-free and (HY) hybrid networks, when attempting to learn to interpret 200 input signals. Mean of 100 samples.

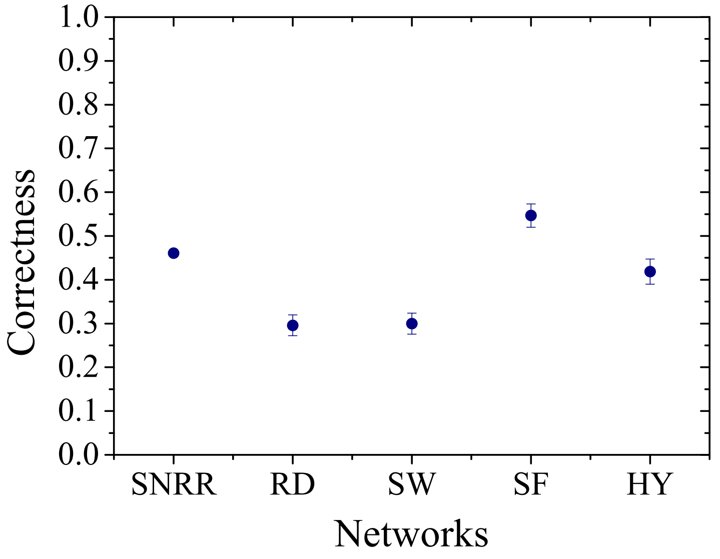

Figure 2. Learning curves of the semantic network based on the Rules of Robotic and the random, small-world, scale-free and hybrid artificial neural networks.

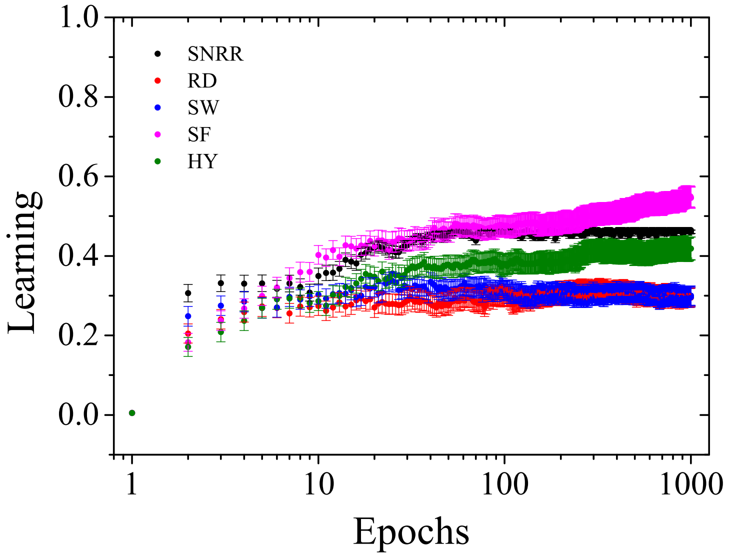

References

[1] Asimov, I. I, Robot. New York: Gnome Press, 1950.
